# Challenges of Integrating New Technologies for Orthopedic Doctors to Face up to Difficulties during the Pandemic Era

**DOI:** 10.3390/healthcare11111524

**Published:** 2023-05-23

**Authors:** Marius Niculescu, Octavia-Sorina Honțaru, George Popescu, Alin Gabriel Sterian, Mihai Dobra

**Affiliations:** 1 Faculty of Medicine, “Titu Maiorescu” University of Bucharest, 031593 Bucharest, Romania; mariusniculescu@yahoo.com; 2 Colentina Hospital, Șoseaua Ștefan cel Mare 19-21, 020125 Bucharest, Romania; 3 Faculty of Sciences, Physical Education and Informatics, University of Pitesti, Târgul din Vale 1, 110040 Arges, Romania; 4 Department of Public Health Arges, Exercitiu 39 bis, 110438 Arges, Romania; 5 Emergency Clinical Hospital Dr. Bagdasar-Arseni, Șoseaua Berceni 12, 041915 Bucharest, Romania; 6 Emergency Hospital for Children Grigore Alexandrescu, 30-32 Iancu de Hunedoara Boulevard, 011743 Bucharest, Romania; gabriel.sterian@umfcd.ro; 7 Department of Pediatric Surgery and Orthopedics, University of Medicine and Pharmacy “Carol Davila” Bucharest, 020021 Bucharest, Romania; 8 Center of Uronephrology and Renal Transplant Fundeni, University of Medicine and Pharmacy “Carol Davila” Bucharest, 020021 Bucharest, Romania; dobra.medical@gmail.com

**Keywords:** orthopedics, new technologies, pandemic era, quantitative research

## Abstract

In the field of orthopedics, competitive progress is growing faster because new technologies used to facilitate the work of physicians are continuously developing. Based on the issues generated in the pandemic era in this field, a research study was developed to identify the intention of orthopedic doctors to integrate new medical technologies. The survey was based on a questionnaire that was used for data collection. The quantitative study registered a sample of 145 orthopedic doctors. The data analysis was performed based on the IBM SPSS program. A multiple linear regression model was applied, which analyzed how the independent variables can influence the dependent variables. After analyzing the data, it was observed that the intention of orthopedic doctors to use new medical technologies is influenced by the advantages and disadvantages perceived by them, the perceived risks, the quality of the medical technologies, the experience of physicians in their use, and their receptivity to other digital tools. The obtained results are highly important both for hospital managers and authorities, illustrating the main factors that influence doctors to use emergent technologies in their clinical work.

## 1. Introduction

Since the introduction of mechanization in the first industrial revolution, technology and innovation have been heavily used in healthcare. Industry 4.0, by integrating the Internet and new technologies (e.g., communication technology (ICT), digitization, artificial intelligence (AI), Internet of Things (IoT), cloud technology, cloud computing, known additive manufacturing (AM), and Big Data and cyber systems), initiated a “paradigm shift” in how patient care is delivered globally across all specialties, including orthopedics [1,2].

In the orthopedic field, new technologies help physicians in the early diagnosis of various conditions, the faster development of innovative treatments, and the perioperative surveillance of patients. The overall goal of technologies in orthopedic surgery is to improve surgical accuracy and achieve faster postoperative rehabilitation, as well as to provide optimized services for patients [3]. Technological innovations have a very important role in preoperative surgical planning, the precise intraoperative placement of implants, the restoration of biomechanical parameters, and the efficient execution of orthopedic procedures. In trauma care, computer-assisted virtual reality, 3D printing techniques for planning the surgical management of acetabular fractures, real-time navigation, and computer-guided and robot-assisted surgery (RAS) applications have grown exponentially in total hip arthroplasty and in tumor reconstruction surgery [4,5,6].

Self-monitoring and reporting technology (SMART) and sensor-based implants have contributed to improved diagnoses provided by physicians, objective intraoperative assessment of soft tissue balance in total knee arthroplasty, and postoperative patient monitoring [7]. Studies have illustrated that advanced technological rehabilitation in hospitals and at home using wearable or implantable sensors is the future [8]. However, there is debate regarding the cost-effectiveness of such an intervention given the need for a steep learning curve and universal applicability in healthcare in underdeveloped countries [7].

Additive manufacturing (AM), also known in the specialized literature as three-dimensional (3D) printing, represents the process of joining materials to create objects from a 3D digital model layer by layer [9]. For many years, this technology has been used in many industries such as in the realm of jewelry making, engineering, fabrics, etc. [9]. Chuck Hall created stereolithography (STL) in 1984 [10]. Since then, this technology has become more and more used due to its versatility, ease of use, and precise control in terms of the manufacturing process but also in terms of the possibility of making complex shapes and structures. Therefore, the printed patterns have the potential to intentionally possess properties that are highly sought after for biomedical applications [9]. In the medical field, AM technology is used to create customized medical instruments, drug delivery systems, engineered tissues, scaffolds for bone regeneration, prosthetic sockets, orthoses, or guides as well as surgical implants [11]. In the last decade, there has been an increasing trend towards the customization of business models and technological advances, which has led to the reduction in costs and expertise required in the exploitation of AM [12]. “Computer-aided design” (CAD) also began to be used in the medical field, being seen as a process of using computer software to help facilitate the activities carried out by medical personnel [13].

There are various techniques for measuring and modeling existing objects (including the human body) to create digital models that we can work on using CAD software. The most used methods include computed tomography (CT) and 3D scanning [11]. Three-dimensional scanning is the most practical and comfortable solution to capture topography. There is a lot of affordable hardware and software, the training requirement is minimal, and it can be very effective [14]. Acquisition time and spatial resolution differ greatly between 3D scanners, ranging from a few minutes to longer and from 0.1 mm to several millimeters [15,16]. The most used systems are laser techniques and structured light methods. The laser technique uses a handheld device to project a laser beam onto the surface, while a sensor measures the distance to the projector.

Digital reconstruction, CAD modeling, and conversion to STL format are performed with appropriate software, with several free alternatives available on the market [17,18]. In the biomedical field, the use of AM technology is increasing, being particularly widespread in the manufacture of orthoses. However, it is still a relatively new approach, with orthotics starting to be 3D printed less than a decade ago. The manufacture of orthoses and prostheses is still largely manual and because of this, the result depends on the human resource involved [11].

The advantages of orthoses made with the help of AM technology are the following: production is possible at lower costs, the possibility of making changes more easily, and faster manufacturing. Patients usually feel more comfortable with prosthetic sockets made with AM appliances than with traditional, hand-made sockets [11,19]. AM technology has been used to facilitate the manufacture of orthoses in a number of situations, namely spinal orthoses, knee orthoses, ankle-foot orthoses, wrist orthoses, foot orthoses, chronic pain relief, or peripheral nerve damage. Custom wrist orthoses for chronic wrist pain [20] or for splinting a healing fractured bone can also be made using AM technology. A recent literature review evaluated a 3D-printed wrist orthosis for a Colles fracture [21]. Radiographs of the wrist were performed periodically to observe both the angle of inclination of the palm, the angle of cubital deviation, and the height of the beam.

Three-dimensional technology is increasingly used in orthopedics. An exponential growth of digital applications in this field of activity is expected in the coming years. Computer technologies play a crucial role in the orthopedic field. Until recently, surgical planning was typically performed manually on fluoroscopic images. These are currently being replaced by advanced planning software that incorporates multimodal and patient-specific medical data. In addition to pre-operative planning, digital technologies have begun to increasingly support the work carried out by doctors. For example, during arthroplasty procedures, computer-assisted techniques [22] have been shown to be superior to conventional implantation techniques in terms of both their consistency and accuracy [23,24,25,26,27].

Robotic solutions are used in many fields to reduce human error, increase accuracy and ensure reproducibility [28,29,30,31]. However, they are still not widely adopted clinically in different disciplines. The main disadvantages of robotic solutions in surgery refer to their intellectual and haptic adaptive behavior, which is minimal; the existence of limitations in terms of integrative interpretation and action in complex situations; poor patient registration; complex configuration; invasive fiducial implantation; and interrupting the workflow [30].

While robotic technologies are mainly aimed at supporting doctors with precise and planned mechanical actions, Augmented Reality (AR) technology enhances the surgeon’s work by increasing the medical information available. AR refers to the real world augmented with virtual information, as opposed to Virtual Reality (VR), where the user experiences a completely virtual setting [32,33]. The growing interest in AR in orthopedics and trauma is not new. Surgical procedures in orthopedic surgery frequently use visual data, such as medical images obtained both pre- and intra-operatively, and often include mechanical steps, such as the screw or implant. Therefore, such technical tasks intensify the use of AR in this field.

Artificial intelligence-based techniques have significantly contributed to the improvement of medical imaging through data acquisition, reconstruction, analysis, and interpretation [34]. Artificial intelligence identifies the imaging examination that the patient needs by incorporating information extracted from the patient’s medical record and determines the appropriate protocol for it [35]. Artificial intelligence can also increase data acquisition speed in nuclear magnetic resonance (NMR) imaging and reduce radiation dose in computed tomography (CT) [36]. The area where artificial intelligence is most used is image interpretation, where AI is used to support radiologists in data interpretation, avoid human errors, and increase diagnostic accuracy.

### 1.1. How AI Works in Analyzing Medical Datasets

AI-based algorithms have been used to recognize the arthroplasty component on plain radiographs, thereby providing a set of images and key imaging features that a radiologist would normally use to distinguish between different types and brands of implants [37]. In this way, the AI-based system tries to match the known characteristics with the parameters of the implant, thus making an assessment. After this process is completed, the accuracy of the system is established by comparing the established and known (correct) results, and inaccuracies or additional information can be manually entered into the system, and thus the process can be repeated. With the help of computing power and algorithm refinement capability, the AI system can learn to self-assess its performance and improve by modifying its internal algorithmic codes [38].

To maximize the predictive power, the AI index algorithm is increasingly refined after being exposed to training datasets, which leads to improved iteration accuracy [39]. This case requires completely different iterative programming, where the program has the autonomy to write its own coding instructions and is thus a step forward towards achieving full automation, this process is called Deep learning (DL) [40,41,42,43].

Deep learning (DL) eliminates the need for human operator input in the algorithm refinement process, thereby reducing the time to achieve a viable system. The algorithmic performance of modern DL neural networks allows the artificial foundation of multi-layered “evolutionary plexuses”, just like the neural system of the human brain [44,45]. Most deep learning systems incorporate some form of artificial neural network (ANN), having a series of iterative processing steps between the input and output layers [46,47,48].

### 1.2. The Use of New Technologies in Orthopedic Practice

In orthopedic practice, the computer has the role of processing data sets belonging to several patients. By means of specialized coding, it has the ability to recognize specific patterns [46,48,49]. Computer software based on artificial intelligence can significantly contribute to the management of patient information. Before using artificial intelligence to fully manage data, orthopedic doctors must understand the technological potential of this technology in the healthcare system. Artificial intelligence algorithms have been used in orthopedics, including for the diagnosis of fractures and osteoarthritis and for establishing bone strength [34].

Some studies have illustrated that, in the orthopedic field, AI has the ability to detect wrist fractures as well as spinal compression on radiographs more easily and quickly (as opposed to medical specialists) [34,50]. Artificial intelligence can help automate the grading of lumbar disc pathology on MRI using different grading systems with nearly 100% accuracy [51]. AI enables automatic segmentation of the area of interest, thus increasing the quality of image analysis, with many studies focusing on the segmentation of knee cartilage [52]. AI-based image interpretation can be very accurate, but requires large training data sets, which are expensive.

A benefit offered by AI in the medical field refers to the possibility of predicting clinical outcomes for patients based on data sets as well as medical imaging. Assessing risks and predicting outcomes has always been a challenge in clinical medicine. In orthopedics, ML can be used in patient management, providing a patient-specific postoperative complication rate after the surgery is performed [53]. Artificial intelligence can increase the accuracy of diagnosis, this aspect mitigating the risk of misdiagnosis. In this way, doctors have the ability to determine the correct medical procedure for each patient.

Currently, AI-based systems have been used in various ways, such as in the identification of fractures and osteoarthritis, the identification of bone mineral density, and the assessment of bone age [34,54]. The activity of orthopedic doctors depends to a high extent on the imaging examination performed, so they can provide a correct diagnosis and treatment only after analyzing the images in question. In this case, artificial intelligence can optimize the acquisition, reconstruction, analysis, and interpretation of images, thus providing effective help to orthopedic doctors [55,56,57].

### 1.3. The Use of Robots in the Orthopedic Field

In recent years, robots [58] have started to be used more and more in the medical field. AI technology can help orthopedic doctors in the diagnostic process. An example of technology that can be used in this regard is the IBM Watson Health cognitive computing system that uses ML to create a cancer treatment support system with the intention of improving diagnostic identification. Orthopedic surgery began to incorporate robotic technologies in 1992 with the introduction of ROBODOC for total hip replacement [59]. This is an active-autonomous, image-based robotic system that has assisted surgery in cementless total hip arthroplasty (THA) [60]. This technology has not been very successful due to the complexity and long duration of the surgical procedure [61].

Another early image-guided autonomous robotic system was CASPAR, used for THA and total knee arthroplasty. To date, no major adverse effects related to the use of CASPAR have been reported [62]. RIO (Robotic Arm Interactive Orthopedic System) is another robotic system that requires the active participation of the doctor in the knee arthroplasty procedure. This robotic system generates a 3D model of the patient’s anatomy that helps the doctor develop a preoperative plan. Most orthopedic surgery robots such as RIO are used for knee or hip arthroplasty [63].

The robotic arm has the ability to assist doctors in performing minimally invasive surgical interventions, offering them other advantages such as three-dimensional imaging, 7 degrees of articulation, and the possibility of operating remotely. An example of this is the TianJi robot. This is an orthopedic field robot that can perform whole spine (spinal instrumentation), pelvis, and knee procedures [64,65]. The robot works through a robotic arm that uses a real-time navigation system with a high degree of precision. Machine learning has various uses in the orthopedic field, such as fracture detection, bone tumor diagnosis, mechanical loosening of hip implants, and osteoarthritis grading [66]. The importance of AI and ML in the orthopedic field is in a constant process of growth, in line with the evolution of research in the field.

In 2019, the first remote multicenter orthopedic surgery using 5G technology was performed by Professor Wei Tian. Robots have started to be used more and more in recent years, especially in surgery due to their precision in performing operations with reduced risks of bleeding in a shorter time. Currently, the use of robots in the operating room is still in an early stage of development; however, there is increased interest among medical and research professionals. Although several robots have already been used to perform surgeries, there is insufficient evidence of their effectiveness over time. In theory, robotic systems are mainly used to prevent misdiagnosis and massive bleeding and to reduce the burnout of physicians in this field.

The COVID-19 pandemic has influenced to a very high extent the activity carried out by physicians. They had to treat patients in special conditions, and very often they had to use digital technologies to get in touch with patients who are far away. Orthopedic specialists also had to face the existing pressures during the period of COVID-19. The complications that arose due to contact with the new virus put the medical staff in a difficult position and because of this they had to resort to additional solutions to face these difficulties. New technologies have facilitated the work of specialists, facilitating the performance of surgical interventions. Thus, physicians had the opportunity to save time and financial resources in carrying out these interventions. Currently, in Romania, new medical technologies are used in a rather low proportion within the state health facilities. However, there are some private clinics or hospitals that benefit from advanced technological equipment, which allows physicians to thoroughly investigate certain cases and provide appropriate treatments in a much shorter time.

Considering the multitude of medical technologies currently existing in the field of orthopedics as well as their role in facilitating the activities carried out by medical personnel, we considered it necessary to carry out quantitative research aimed at identifying the intention of orthopedic doctors to use the new medical technologies in the activities they carry out in this pandemic era. In this study, a multiple linear regression model was carried out. The independent variables that were taken into account at the level of this multiple linear regression model were previously analyzed at the level of other specialized studies. These were the following [67,68,69,70,71,72,73,74,75]: ease of use, promotional activities carried out regarding new medical technologies, advantages perceived by orthopedic doctors regarding new medical technologies, physicians’ experience in using these medical technologies, their receptivity regarding the use of new medical technologies, the quality of existing new medical technologies in the field of orthopedics, the disadvantages perceived by physicians regarding these technologies, the reliability in time of new medical technologies, and the risks perceived by orthopedic doctors regarding the use of new medical technologies, as well as the acquisition cost of new medical technologies.

The main aim of this study was to identify the degree of use of the new technologies in orthopedics and the receptivity of orthopedic doctors to use the new medical technologies in this field. The secondary aims established at the level of this research were to identify the perception of the doctors regarding the importance of the new technologies in the field in which they practice; to identify the sources of information used by the respondents to inform themselves about the new technologies; to identify how often the orthopedic doctors are using the new medical technologies within the medical units; to identify the types of medical technologies that the respondents would like to use in their daily activities; and to identify the main benefits obtained by the doctors following the use of the new medical technologies in this field.

### 1.4. The Research Hypotheses Were the Following

**H1.**  *The easy use of new medical technologies on the market has the ability to directly and positively influence the intention of orthopedic doctors to use these technologies in their work.*

**H2.**  *Promotional activities carried out for new medical technologies have the ability to directly and positively influence the intention of orthopedic doctors to use these technologies in their work.*

**H3.**  *The advantages perceived by orthopedic doctors regarding new medical technologies have the ability to directly and positively influence their intention to use these technologies in their work.*

**H4.**  *Orthopedic doctors’ experience in using new medical technologies has the ability to directly and positively influence their intention to use these technologies in their work.*

**H5.**  *Orthopedic doctors’ receptivity to the use of new medical technologies has the ability to directly and positively influence their intention to use new medical technologies in their work.*

**H6.**  *The quality of new medical technologies has the ability to directly and positively influence the intention of orthopedic doctors to use these technologies in their work.*

**H7.**  *Disadvantages perceived by orthopedic doctors regarding new medical technologies have the ability to directly and negatively influence their intention to use new medical technologies in their work.*

**H8.**  *The reliability of new medical technologies in the field of orthopedics has the ability to directly and positively influence the intention of orthopedic doctors to use these technologies in their work.*

**H9.**  *The risks perceived by orthopedic doctors regarding new medical technologies have the ability to directly and negatively influence their intention to use these technologies in their work.*

**H10.**  *The acquisition cost of new medical technologies in the field of orthopedics has the ability to directly and negatively influence the intention of orthopedic doctors to use these technologies in their work.*

This research provides information regarding the main variables that underlie the decisions of orthopedic doctors regarding the use of new medical technologies. The results provide clear information regarding the main factors behind the intention of orthopedic doctors to use these technologies in the private or state clinics where they work.

## 2. Materials and Methods

### 2.1. Survey Design

The data collection was carried out with the help of a questionnaire that was later distributed to the respondents. Regarding the way in which the research instrument was created, it should be stated that the first question at its level was a filter, with the role of selecting only orthopedic doctors who work in a clinic or in a hospital in Romania. Later, there were several questions through which it was desired to identify the opinion of physicians regarding the usefulness of new medical technologies in the field of orthopedics. In the last part of the questionnaire, several questions were used to identify information regarding the orthopedic doctors who participated in this study and the activities they carry out. Regarding the type of scale used to measure the link between the variables, it should be noted that the 10-point Likert scale was used. The sampling method used was that of the snowball. Regarding the sample, the quantitative study was carried out on 145 orthopedic doctors in Romania. The research was carried out between December 2022 and January 2023.

Analyzing from the perspective of the respondents’ profile, it should be stated that, of the total of 145 respondents who participated in the study, most of them (38.6%) were between 36 and 45 years old and 33.1% of them were between 25 and 35 years old, while 20% of the orthopedic doctors who participated in the study were aged between 46 and 55 years. A smaller proportion of those who participated in the research (8.3%) stated that they were over 55 years old. Regarding the gender distribution of the respondents, the majority of those who participated in the study (90.3%) were men, while 9.7% of them were women. A total of 29% of orthopedic doctors worked in a hospital or clinic in Bucharest or Ilfov, while 71% of them worked in another health facility in the country. Studying from the perspective of the type of health facility in which the respondents work, 50.3% of them stated that they worked both in a state hospital and in a private health facility, 28.3% of them only worked in a state health facility, while 21.4% of them worked in a private clinic (Table 1).

### 2.2. Regression Model

The regression model that was carried out at the level of this study had the role of determining the link between the dependent variable (the intention of orthopedic doctors to use the new medical technologies in the pandemic era) and the independent variables: the ease of use, the promotional activities carried out regarding the new medical technologies, the advantages perceived by the orthopedic doctors regarding the new medical technologies, the experience of physicians in using these medical technologies, their receptivity regarding the use of the new medical technologies, the quality of the new medical technologies existing in the field of orthopedics, the disadvantages perceived by physicians regarding these technologies, the reliability over time of new medical technologies, the risks perceived by orthopedic doctors regarding the use of new medical technologies as well as the acquisition cost of new medical technologies.

The following formula was used to create the linear multiple regression model [76]:

Y = β0 + β1 ∗ X1 + β2 ∗ X2 + β3 ∗ X3 + β4 ∗ X4 +...+ βn ∗ Xn + Ɛ
(1)

Within it, the following indicators can be found: Y (the dependent variable at the level of the linear multiple regression model), the β coefficients, the constant (β0), the standard error (Ɛ) as well as the model parameters (X). Applying the previous formula to the model made in this study, the following emerge:
The intention of orthopedic doctors to use the new technologies in the medical field=β0 + β1 ∗ Ease of use of new medical technologies + β2 ∗ Promotional activities carried out regarding new medical technologies + β3 ∗ The advantages perceived by orthopedic s doctors regarding the new medical technologies + β4 ∗ The experience of physicians in the use of these medical technologies + β5 ∗ The receptivity of orthopedic doctors regarding the use of new medical technologies + β6 ∗ The quality of the new medical technologies existing in the field of orthopedics + β7 ∗ The disadvantages perceived by physicians regarding the new medical technologies + β8 ∗ Reliability over time of new medical technologies + β9 ∗ The risks perceived by orthopedic doctors regarding the use of new medical technologies + β10 ∗ The acquisition cost of new medical technologies + Ɛ


## 3. Results

In the first part of the quantitative study, the opinion of orthopedic doctors regarding the new medical technologies existing on the market as well as their role in the pandemic era was analyzed. Following the analysis and interpretation of the results, it was noticed that orthopedic doctors believe that these technologies are very important in the field in which they practice. The main sources of information that they use to document themselves about the new technologies appearing on the market in the field of orthopedics are internships abroad (28.3%), specialized publications (13.8%) as well as profile websites where detailed information is presented regarding the advantages offered by new technologies, and the costs incurred for them as well as their usefulness in this field (13.8%). A total of 12.4% of the physicians who participated in the study stated that they obtained information about these technologies from the national and international symposia they attended, 9.7% of them stated that they read existing articles on social networks, while 5.5% of them participated in various online courses. A total of 16.6% of the physicians stated that they documented from other sources (Table 2).

The specialists in orthopedics who participated in the study stated that, currently, within the health facilities where they mainly carry out their activity, they use the new medical technologies to a small extent. Only 15.9% of those who participated in the study stated that they had used the new technologies in the field so far, while a large part of them (84.1%) stated that they did not have this opportunity but would want to be able to use them in the next period of time. Regarding the technologies that the respondents would like to use later, most of them (42.1%) stated that they would like to use technologies based on artificial intelligence, 28.3% of them mentioned that they would like to carry out investigations based on a series of robots, 12.4% of those who participated in the study would like to work with technologies based on 3D Printing technology, 9.7% of them would like to use IoT technology for solving the problems they encounter, while 7.6% of the respondents would like to use augmented reality (Table 3).

Regarding the main advantage of the new medical technologies that can be used in orthopedics, in Table 4, it can be seen that 31.7% of the orthopedic doctors who participated in the study stated that they facilitated the work carried out by the medical staff. A total of 30.3% of them specified the fact that these technologies have the ability to provide detailed information about various conditions that patients suffer from, 26.9% of them considered that reducing the execution time of certain surgical interventions is the most important advantage, 7.6% of them believed that they had the role of reducing costs, while 3.4% of them believed that the higher success rate obtained from the use of these technologies was their main benefit.

Analyzing the results obtained at the level of the linear multiple regression model, it can be seen that the value of R is 0.944, while the value of R Square is 0.890, which illustrates the fact that 89% of the variation of the dependent variable is explained by the independent variables considered at the level of this model. The value of Adjusted R Square is 0.882 while the standard error registers the value of 0.584 (Table 5).

Following the analysis, it was noticed that the value of F is 108,850, and the value of the degrees of freedom is 10 (df1), respectively, 134 (df2). The value of Mean Square is 37,061. Since the value of Sig. < 0.05, the proposed model is accepted (Table 6).

Analyzing the table of coefficients, in Table 7, it can be seen that only 6 of the 10 independent variables that were considered at the level of the linear multiple regression model were accepted, registering a value of Sig. < 0.05. The independent variables that can be included in the regression model are the advantages perceived by orthopedic doctors with regard to the new medical technologies, the experience of physicians in using these medical technologies, their receptivity to the use of the new medical technologies, the quality of existing new medical technologies in orthopedics, the disadvantages perceived by physicians regarding these technologies as well as the perceived risks. The four variables for which the value of Sig. was greater than 0.05 are the ease of use of the new medical technologies (Sig. = 0.867), the promotional activity carried out for these medical technologies (Sig. = 0.349), the reliability of the new medical technologies (Sig. = 0.056) as well as the cost of acquisition of the new technologies (Sig. = 0.476).

Applying the indicators obtained in Table 5 (Std. Error of the Estimate) and in Table 7 (β coefficients and the constant) within Formula (1) of the multiple linear regression model, the following formulas are obtained:

The intention of orthopedic doctors to use the new technologies in the medical field = 0.316 ∗ The advantages perceived by orthopedic doctors regarding the new medical technologies + 0.307 ∗ The experience of physicians in the use of these medical technologies + 0.254 ∗ The receptivity of orthopedic doctors regarding the use of the new medical technologies + 0.351 ∗ The quality of the new medical technologies existing in the field of orthopedics − 0.80 ∗ The disadvantages perceived by physicians regarding the new medical technologies − 0.76 ∗ The risks perceived by orthopedic doctors regarding the use of the new medical technolo-gies + 0.584

## 4. Discussion

The new technologies appearing on the market have greatly improved both the medical and pharmaceutical fields [77]. They facilitated the work carried out by physicians, the rapid identification of diagnoses, the performance of certain medical procedures, and communication with the patients [78]. Some technologies have helped physicians to more easily determine the most suitable treatment, perform surgical interventions as precisely as possible, and monitor permanent patients [79]. Their role is to quickly search databases and provide quick answers to certain problems that would normally take a very long time. These medical technologies have the role of reducing human error and limiting problems that may arise due to mistakes made by physicians or nurses [80]. The emergence of IoT, artificial intelligence, 3D technologies [81], or robots has revolutionized medical activities, with these technologies having the role not to replace the medical personnel, but to support their activity. The current capabilities of automatic and deep learning are not yet ready for a fully autonomous surgical intervention, as they are highly dependent on the human resource performing a real-time task on the patient. Those who support the use of robots on an increasingly large scale appreciate the benefits of robotic systems in performing minimally invasive surgical interventions, although they are aware of the costs and the lack of clear benefits that they bring.

The lack of awareness of the capability of computing power as well as the complex nature of interaction with human tissue has led to delays in the penetration of AI in orthopedic surgery. However, artificial intelligence is advancing at a rapid pace, its role being to support medical practice and not to remove specialists from this field. The benefits of implementing robots in orthopedics cannot be guaranteed, as in-depth studies need to be conducted to analyze in detail the connection between intelligent computers, the patient, and the doctor. Future robots could provide a degree of precision that would not be possible to achieve by human specialists [82]. The environment could also be controlled with AI, such as table tilt, temperature, and lights.

In this paper, quantitative research was carried out that aimed to identify the intention of orthopedic doctors to use the new technologies. The results illustrated that orthopedic doctors consider the new technologies in the medical field to be of major importance in the activities that they carry out, gathering information about them both from internships abroad as well as from specialized publications or websites. In the future, they would like to use advanced devices based on artificial intelligence, robots, or 3D printing technology. Regarding the linear multiple regression model, it was noticed that the intention of orthopedic doctors to use the new medical technologies in this field of activity is influenced in a high proportion by the advantages perceived by orthopedic doctors, by their experience regarding the use of the new technologies, by their receptivity regarding their use, the quality of the medical technologies existing on the market, the disadvantages perceived by orthopedic doctors, but also the risks perceived by them.

Regarding the limits of this quantitative study, it must be stated that it was carried out on a number of 145 respondents, which does not allow us to generalize the results to the entire researched community. Moreover, at the level of quantitative research, only the impact that 10 independent variables have on the dependent variable (the intention of orthopedic doctors to use the new medical technologies in the pandemic era) was studied. In the future, these studies should be carried out starting with certain in-depth interviews to be carried out with several experts in the field of orthopedics. These interviews can provide detailed information regarding the opinion of physicians regarding the possibility of using new medical technologies in this sector of activity. In addition, they can be used to identify other independent variables that can be considered at the level of the linear multiple regression model. Moreover, in the future, other studies should be carried out both among hospital managers and among those who coordinate the activity carried out within private clinics. Their aim should be to identify the barriers they encounter in acquiring the new technologies as well as how the acquisition process of new technologies can be facilitated so that orthopedic doctors have the opportunity to work with such medical tools in the future.

## 5. Conclusions

The use of new medical technologies in the field of orthopedics is not limited only to ML, DL, NLP, and computer vision, researchers in this field aim to achieve completely autonomous medical actions. Even if they are being carried out to a certain extent at the moment, it is desired to apply them on a much wider scale. Orthopedic doctors should be prepared for the use of the new technologies, there should be specialized training to show them how they can be implemented in the operating room or even in the practice. In the future, it is considered that they will be used more and more efficiently in the decision-making process and human risks and errors will be eliminated. Orthopedic doctors can help integrate new technologies into modern medical practice, and because of this, they should constantly collaborate with scientists, providing them with the data they need to implement them in various applications. The new technologies can revolutionize the field of orthopedics and have the ability to provide optimized patient care in the near future. In the future, these technologies are expected to reduce physician burnout as well as the time required to analyze a particular case or perform a surgical intervention, thus allowing for an increase in the number of appointments or surgeries performed per day.

## Figures and Tables

**Table 1:** Presentation of the respondents’ profile.

Category		Frequency	Percentage (%)
Gender	Male	131	90.3
	Female	14	9.7
Age	25–35	48	33.1
	36–45	56	38.6
	46–55	29	20
	Over 55	12	8.3
Place where the physicians practice	In Bucharest-Ilfov	42	29
	In a different county in the country	103	71
Health facility where the respondents work	State	31	21.4
	Private	41	28.3
	Both in a state health facility and in a private one	73	50.3

**Table 2:** The sources of information used by the respondents to inform themselves about the new technologies on the market in the field of orthopedics.

Sources of Information Used	Frequency	Percentage (%)
Symposia	18	12.4
Specialized publications	20	13.8
Specialized sites	20	13.8
Social networks	14	9.7
Internships abroad	41	28.3
Courses held online	8	5.5
Others	24	16.6

**Table 3:** The new technologies that orthopedic physicians would like to use in the next period of time.

Technologies	Frequency	Percentage (%)
Iot	14	9.7
Artificial intelligence	61	42.1
Robots	41	28.3
3D printing	18	12.4
Augmented Reality	11	7.6

**Table 4:** Respondents’ perception of the main benefit obtained by orthopedic doctors following the use of the new medical technologies in this field.

Technologies	Frequency	Percentage (%)
They facilitate the activity of medical specialists	46	31.7
They reduce the performance time of some interventions	39	26.9
They reduce the costs of carrying out some interventions	11	7.6
Higher success rate with certain treatments	5	3.4
They provide detailed information on specific conditions	44	30.3

**Table 5:** Model summary.

R	R Square	Adjusted R Square	Std. Error of the Estimate	R Square Change
0.944	0.890	0.882	0.584	0.890

**Table 6:** ANOVA analysis.

Model	F	df	Sum of Squares	Mean Square	Sig.
Regression	108.850	10	370.610	37.061	0.000
Residual		134	45.624	0.340	
Total		144	416.234		

**Table 7:** Table of coefficients.

	Unstandard. Coeff.		Stand. Coeff.	t	Sig.	Correlations			Collinearity Statistics	
	B	Std. Error	Beta			Zero-order	Partial	Part	Tolerance	VIF
(Constant)	−0.086	0.281		−0.308	0.759					
The ease of use of the new medical technologies	0.005	0.028	0.006	0.168	0.867	0.231	0.015	0.005	0.677	1.477
The promotional activities carried out	0.022	0.024	0.031	0.939	0.349	0.228	0.081	0.027	0.750	1.333
The perceived advantages	0.240	0.031	0.316	7.694	0.000	0.751	0.554	0.220	0.486	2.058
The experience of physicians in the use of the new technologies	0.259	0.036	0.307	7.121	0.000	0.776	0.524	0.204	0.440	2.272
The receptiveness of physicians in the use of the new technologies	0.222	0.038	0.254	5.912	0.000	0.746	0.455	0.169	0.442	2.261
The quality of the new technologies	0.289	0.029	0.351	10.064	0.000	0.676	0.656	0.288	0.673	1.487
The disadvantages of the new technologies	−0.060	0.025	−0.080	−2.409	0.017	0.305	−0.204	−0.069	0.739	1.354
The reliability of the new technologies	0.055	0.028	0.073	1.926	0.056	0.498	0.164	0.055	0.573	1.747
The perceived risks	−0.060	0.026	−0.076	−2.275	0.024	0.278	−0.193	−0.065	0.731	1.368
The acquisition cost of the new technologies	−0.020	0.028	−0.026	−0.715	0.476	0.417	−0.062	−0.020	0.613	1.633

## Data Availability

Not applicable.
